# miR199a-5p inhibits hepatic insulin sensitivity via suppression of ATG14-mediated autophagy

**DOI:** 10.1038/s41419-018-0439-7

**Published:** 2018-03-14

**Authors:** Bo Li, Xiangsong Wu, Hanbei Chen, Chengle Zhuang, Zhiguo Zhang, Shuangshuang Yao, Dongsheng Cai, Guang Ning, Qing Su

**Affiliations:** 1 Department of Endocrinology, XinHua Hospital, Shanghai Jiao Tong University School of Medicine, Shanghai, 200092 China; 2 Department of General Surgery, XinHua Hospital, Shanghai Jiao Tong University School of Medicine, Shanghai, 200092 China; 3 Department of Gastrointestinal Surgery, Shanghai Tenth People’s Hospital Affiliated with Tongji University, Shanghai, 310000 China; 4 Shanghai Institute of Endocrine and Metabolic Diseases, Shanghai Clinical Center for Endocrine and Metabolic Diseases, RuiJin Hospital, Shanghai Jiao Tong University School of Medicine, Shanghai, 200025 China; 5 Department of Molecular Pharmacology, Diabetes Research Center, Institute of Aging, Albert Einstein College of Medicine, Bronx, New York 10461 USA

## Abstract

MicroRNAs (miRNAs) are known to contribute to many metabolic diseases, including diabetes. In this study, we investigated the role of miR199a-5p in the regulation of hepatic insulin sensitivity. Ad-anti-miR199a-5p adenoviruses were injected into male C57BL/6J WT mice fed a high-fat diet to inhibit miR199a-5p expression before the glucose levels and insulin resistance were assessed. Similarly, Ad-miR199a-5p adenoviruses were injected into male C57BL/6J WT mice to cause the overexpression of miR199a-5p. To investigate the roles of autophagy-related protein 14 (ATG14) and miR199a-5p in the regulation of insulin sensitivity, we injected Ad-miR199a-5p with or without Ad-ATG14 viruses into WT C57BL/6J mice before performing functional assays. Moreover, we infected HepG2 cells or primary hepatocytes with Ad-anti-miR199a-5p or Ad-miR199a-5p viruses to determine the effect of miR199a-5p on insulin resistance in vitro. Finally, we explored the clinical relevance of miR199a-5p by examining the expression level of miR199a-5p in liver samples derived from diabetes patients. We first demonstrated that knocking down miR199a-5p led to decreased glucose tolerance and clearance in vivo, whereas the overexpression of miR199a-5p had the opposite effect. We further identified ATG14 as the target of miR199a-5p, and ATG14 partially rescued miR199a-5p-potentiated glucose and insulin tolerance. In addition, transmission electron microscopy data and western blot data regarding ATG14, LC3 and BECLIN1 illustrated that miR199a-5p regulates autophagy via ATG14. Knocking down miR199a-5p in primary hepatocytes and HepG2 cells suppressed the insulin-stimulated phosphorylation of insulin receptor β, glycogen synthase kinase 3β and protein kinase B, whereas the overexpression of miR199a-5p further potentiated their phosphorylation. Finally, we detected upregulated miR199a-5p levels, which were correlated with reduced ATG14 mRNA levels and downregulated autophagy in liver samples obtained from diabetes patients. Our study uncovered a novel biological role of miR199a-5p in the regulation of hepatic insulin sensitivity via ATG14-mediated autophagy.

## Introduction

Diabetes, especially type 2 diabetes, has become an alarming global burden. According to the 2015 Global Burden of Disease report, the prevalence of diabetes rose from approximately 333 million patients in 2005 to approximately 435 million patients in 2015, a dramatic 30.6% increase during the last 10 years^1^. Our national cross-sectional survey data demonstrated that the overall prevalence of diabetes in the Chinese adult population was approximately 11.6% in 2010^2^. Among several major pathological changes that occur in type 2 diabetes, insulin resistance is the driving factor, which is characterized as a high blood glucose level, hyperinsulinemia and decreased insulin sensitivity. Because insulin resistance prevents cells from efficiently taking in glucose^3^, the regulation of insulin resistance has attracted considerable attention, and it could be a key component of diabetes treatment.

MicroRNAs (miRNAs), a class of small non-coding RNAs, bind to target mRNAs, destabilizing them and suppressing their translation. miRNAs have been much appreciated as major regulators of gene expression with crucial functions in numerous diseases, including neurological disorders, heart diseases, vascular diseases, viral infections and cancers^4,5^. Recent studies have identified several miRNAs that are associated with the core components of metabolic diseases^6^, such as insulin resistance and lipid metabolism^7–9^.

Among these miRNAs, miR199a-5p, which can be produced from two genes, miR199a-1 and miR199a-2, is an interesting miRNA that has been shown to contribute to lung fibrotic processes, embryonic stem cell-derived hepatic cell repopulation and liver cancer glucose metabolism^10–13^. Moreover, our previous study demonstrated that miR199a-5p is involved in hepatic steatosis^14^. Because of liver’s essential role in regulating lipid and glucose metabolism and a potential link between hepatic insulin resistance and hepatic steatosis^15,16^, we hypothesized that miR199a-5p plays a key role in hepatic insulin sensitivity. Furthermore, several reports have demonstrated the relationship between autophagy and insulin resistance^17–21^. Among the known autophagy-related proteins, autophagy-related protein 14 (ATG14) is the most important regulator involved in autophagic initiation^22^, and we found that ATG14 is an interesting target of miR199a-5p. Therefore, we further proposed a miR199a-5p/ATG14/autophagy/insulin resistance axis in diabetes pathogenesis in our study.

## Materials and methods

### Mouse experiments and human liver tissues

Male C57BL/6 lean mice aged 8–10 weeks originating from the JAX Lab were purchased from the Shanghai Slac laboratory animal company. The mice were housed at 20–24°C with a light/dark cycle of 12 h and fed with either a high-fat diet (HFD) or a standard chow diet as described previously^23^. This animal protocol was approved by the Animal Care Committee of Shanghai Jiao Tong University. To collect human liver samples, patients who were diagnosed as having type 2 diabetes or patients without a diabetes history were enrolled in this study, and all patients signed the written consent form. Liver tissues were collected from patients who underwent regular hepatectomy for hepatic hemangiomas surgeries at the Xinhua Hospital affiliated with the Shanghai Jiao Tong University from 2016 to 2017. This study was approved by the Ethics Committee of Xinhua Hospital affiliated with the Shanghai Jiao Tong University.

### Blood glucose test, serum insulin test, glucose tolerance test (GTT), insulin tolerance test (ITT), pyruvate tolerance test (PTT) and homeostasis model assessment of the insulin resistance index (HOMA-IR)

Blood glucose was measured using a portable blood glucose meter (Roche, ACCU-CHEK Active) using mouse tail vein blood. Serum insulin levels were determined using commercial kits from ALPCO (Salem, NH, USA). GTTs and PTTs were carried out by intraperitoneal injection of 2 g/kg glucose or 1.5 g/kg sodium pyruvate after overnight fasting^9,24^. ITTs were performed by intraperitoneal injection of 0.75 units/kg insulin after 4 h of fasting^9^, and the time points included 0, 15, 30, 60, 90 and 120 min. The HOMA-IR index was first described by Matthews et al.^25^. We calculated the HOMA-IR index based on the following formula: [fasting glucose levels (mM/L)] × [fasting serum insulin level (mU/mL)]/22.5 (ref.^9^).

### Handling recombinant adenoviruses

Six recombinant adenoviruses were customized by Genechem Company (Shanghai, China). Ad-anti-miR199a-5p transcribes reverse miR199a-5p sequences from the vector to silence the expression of miR199a-5p, Ad-anti-miR NC expresses Ad-anti-miR199a-5p’s control scrambled short hairpin RNA, Ad-miR199a-5p overexpresses miR199a-5p, Ad-miR NC expresses Ad-miR199a-5p’s control scrambled short hairpin RNA, Ad-ATG14 overexpresses ATG14, and Ad-NC is Ad-ATG14’s control and expresses green fluorescent protein. Viruses diluted in phosphate-buffered saline (PBS) were administered at a dose of 10^7^ plaque-forming units (PFUs) per well in 12-well plates, or 5 × 10^6^ PFU per well in 24-well plates. For the animal study, 1 × 10^9^ PFU were injected into wild-type (WT) mice, or 2 × 10^9^ PFU were injected into HFD mice via the tail vein^9^.

### Primary hepatocyte isolation, cell culture and treatments

The methods of isolation and culture of primary hepatocytes from C57BL/6J mice were based on methods described previously^26^. HepG2 cells were cultured in Dulbecco’s modified Eagle’s medium (Invitrogen) supplemented with 1% penicillin–streptomycin (Invitrogen), 1% l-glutamine (Invitrogen) and 10% fetal bovine serum (Gibco). 3-Methyladenine (3-MA) was purchased from Sigma-Aldrich.

### Quantitative PCR

The total RNA was extracted from hepatocytes or liver samples by TRIzol reagent (Invitrogen) according to the manufacturer’s instructions. The primer sequences are listed in Supplementary Table 1. Total RNA (1 μg) was transcribed into complementary DNA (cDNA) by Superscript II reverse transcriptase (Invitrogen) and random primer oligonucleotides (Invitrogen). The miRBase accession number for mouse miR199a-5p is MIMAT0000229, and the miRBase accession number of human miR199a-5p is MIMAT0000231. For mouse miR199a-1, the miRBase accession number is MI0000241, and the MGI ID is 2676863. For human miR199a-1, the miRBase accession number is MI0000242, and the HGNC ID is 31571. For mouse miR199a-2, the miRBase accession number is MI0000713, and the MGI ID is 3618742. For human miR199a-1, the miRBase accession number is MI0000281, and the HGNC ID is 31572. To analyze the expression of miR199a-5p, we used gene-specific TaqMan miRNA assay probes (Applied Biosystems, Foster City, CA). Briefly, we reverse-transcribed 1 μg of total RNA to cDNA using AMV reverse transcription (Takara, Kyoto, Japan) and a stem-loop RT primer (Applied Biosystems). The expression levels of mRNA and miRNA were normalized to β-actin and U6 small nuclear RNA, respectively. We performed real-time PCR using a TaqMan PCR kit on the 7900 HT Real-Time PCR System for 40 cycles. We ran all reactions, including controls without templates, in triplicate. For the quantitative PCR analysis, we determined the threshold cycles (CT) values using fixed-threshold settings after the initial cycling was completed. The PCR amplification efficiencies of these primers were between 90% and 110%.

### Western blotting

Homogenized tissues and cells were lysed in RIPA buffer containing 1 × PBS, 1% NP-40, 5 mM EDTA, 0.1% sodium dodecyl sulfate (SDS), 1 mM Na_3_VO_4_, 1% phenylmethanesulfonyl fluoride, complete protease inhibitor cocktail (Sigma) and complete phosphatase inhibitors. The lysates were centrifuged at 12,000 *g* for 10 min at 4°C to remove the insoluble materials, and the supernatants were boiled in SDS loading buffer. The boiled samples were separated by 10% SDS–polyacrylamide gel and electroblotted to nitrocellulose transfer membranes (Whatman, GE Healthcare). The membranes were blocked with 5% milk and incubated with different antibodies, followed by incubation with fluorescent-conjugated secondary antibodies. The primary antibodies used in western blotting included anti-p-IRβ [tyr1162/1163], anti-IRβ (Santa Cruz, Dallas, TX, USA), anti-p-AKT [ser473], anti-AKT, anti-BECLIN1, anti-LC3I/II, anti-p-GSK3β [ser9], anti-GSK3β, and anti-β-actin (Cell Signaling, Beverly, MA, USA) and anti-ATG14 (Proteintech, Chicago, IL, USA).

### Plasmids and luciferase assays

**Fig. 1:**
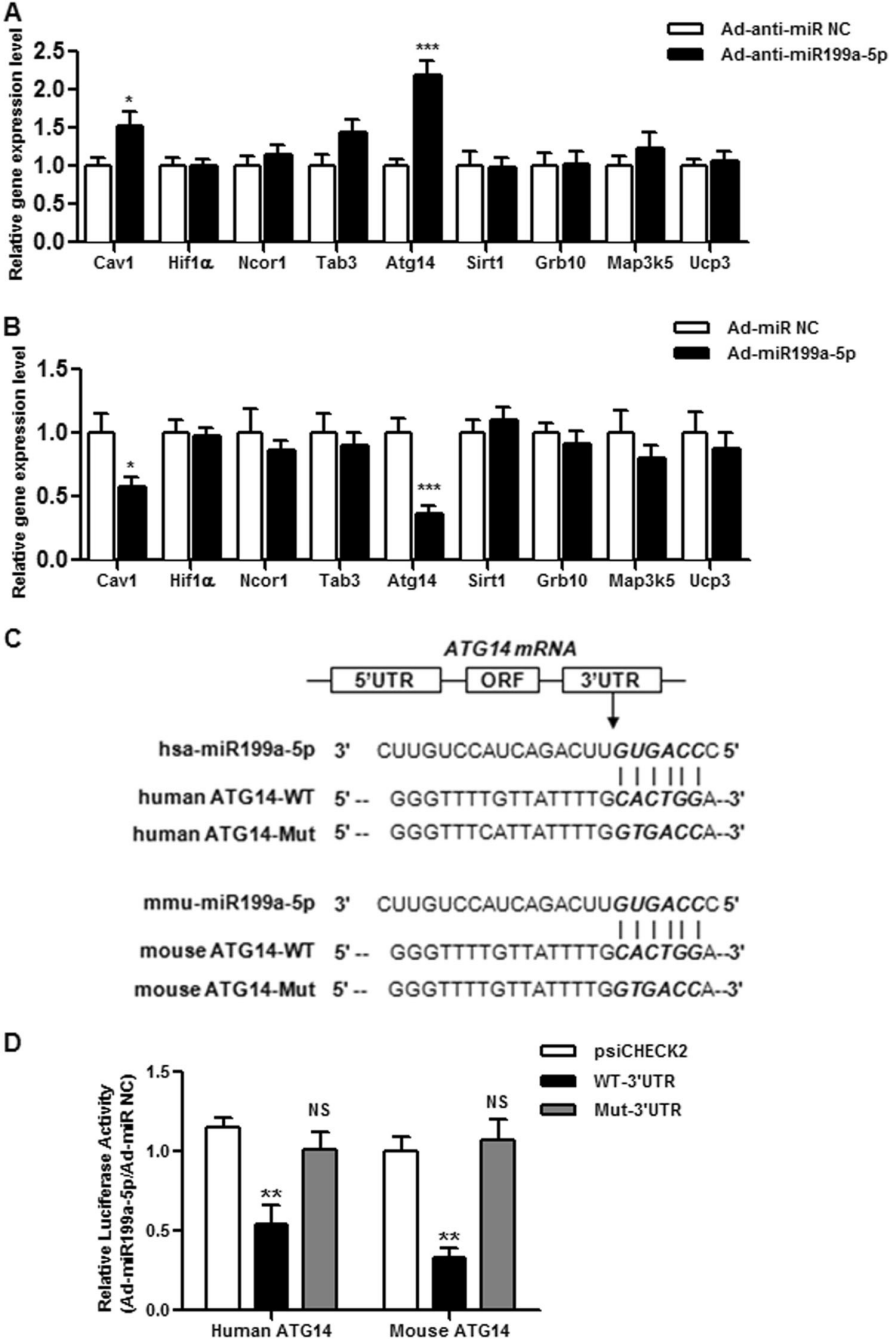
ATG14 is miR199a-5p’s target. **a** The expression profiles of potential targets of miR199a-5p in male HFD-fed C57BL/6J WT mice expressing miR199a-5p. **b** The expression profiles of potential targets of miR199a-5p in male C57BL/6J WT mice overexpressing miR199a-5p. The two-tailed Student's *t-*test was applied to determine the difference between the Ad-miR199a-5p group and the corresponding control or the Ad-anti-miR199a-5p group and the corresponding control (*P < 0.05, ***P < 0.001), *n* = 6-8. **c** Sequence alignment of miR199a-5p within the 3ʹ-UTRs of human and mouse ATG14. The seed region of miR199a-5p is GUGACC. **d** HEK cells were co-transfected with the psiCHECK2-promoter vector and the WT 3ʹ-UTR report plasmid of ATG14 or the mutated 3ʹ-UTR report plasmids of ATG14 (Mut ATG14) with miR199a-5p (Ad-miR199a-5p) or the negative control (Ad-miR NC), and the luciferase signal was determined. The two-tailed Student's *t*-test was applied to determine the difference between the psiCHECK2 group and the WT 3ʹ-UTR group (**P < 0.01) or the psiCHECK2 group and the Mut 3ʹ-UTR group (NS, *P* > 0.05)

The annealed oligonucleotides with the WT ATG14 3ʹ-UTR binding site or the oligonucleotides with mutated miR199a-5p-binding sites (Fig. 1c) were cloned into psiCHECK2 reporter vectors (Promega) between the *XhoI* and *NotI* sites. After being seeded into 24-well plates, HEK293T cells were co-transfected with psiCHECK2 plasmids and Ad-miR199a-5p or the control plasmid using lipofectamine 2000 (Invitrogen). Two days post transfection, luciferase activity was determined by the Dual Luciferase Reporter System (Promega).

### Measurement of autophagic flux by fluorescence microscopy

The method used to measure autophagic flux was described previously^27^. In brief, primary hepatocytes were infected with adenoviruses expressing mCherry and green fluorescent protein (GFP) double-tagged LC3 (Genelily Co., Ltd, Shanghai, China), fixed by 4% paraformaldehyde, and examined with an LSM 510 META confocal laser microscope (Zeiss, Germany). The number of red and yellow punctates in at least 20 transfected cells were counted using Image-Pro Software.

### Transmission electron microscopy (TEM)

Liver tissues were fixed in 2.5% glutaraldehyde in 0.1 M sodium cacodylate buffer (pH 7.4) for 1 h. After being washed three times with 0.1 M phosphate buffer, the cells were fixed with 1% osmium tetroxide in 0.1 M phosphate buffer for another 1 h. The samples were dehydrated with increasing concentrations of ethanol, embedded in epoxy resin and cut to a thickness of 70 nm. Electron photomicrographs were taken of the ultrastructures of the liver tissues with a TEM (Hitachi, Japan), with the imaging parameters of the lens mode set as zoom-1 HC1, acceleration voltage of 80.0 kV, spot size of micro 8 and ×24,500 magnification.

### Statistical analysis

The Kolmogorov–Smirnov test was used to analyze the normal distribution of data, and a two-tailed unpaired Student’s *t*-test was used for the analyses. Means ± SEMs shown are representative of at least three independent in vitro experiments or at least two independent in vivo experiments, and *P < *0.05 was considered statistically significant.

## Results

### Inhibition of miR199a-5p by Ad-anti-miR199a-5p increases insulin sensitivity in vivo

**Fig. 2:**
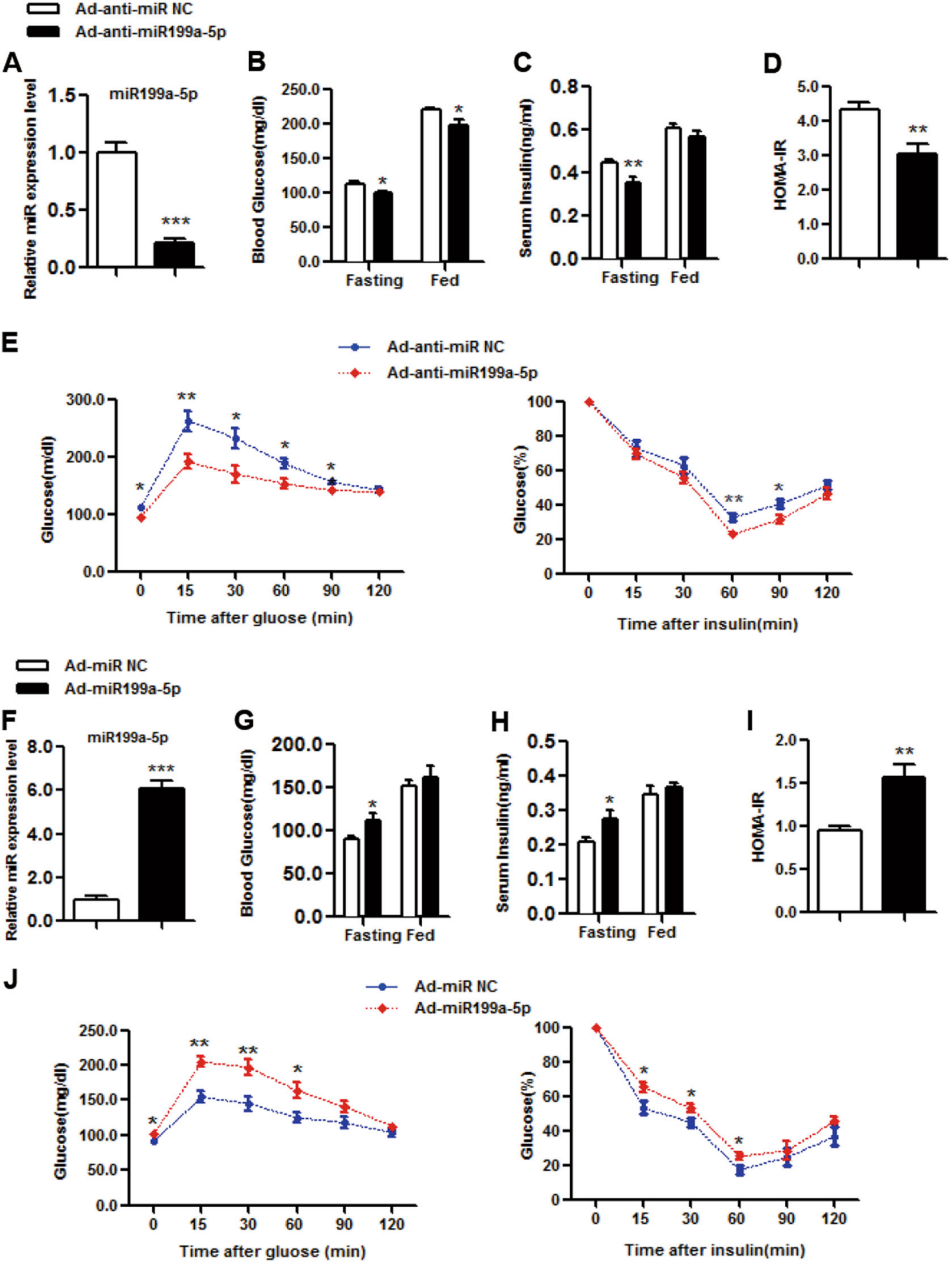
miR199a-5p regulates insulin sensitivity in vivo. **a**-**e** Male C57BL/6J WT mice were fed a high-fat diet for 3 months and then infected with adenovirus-inhibiting miR199a-5p (Ad-anti-miR199a-5p) or the negative control (Ad-anti-miR NC) via tail vein injection. **a** Day 9: measurement of hepatic miR199a-5p expression.** b**, **c** Days 6 and 5: examination of fed or fasting blood glucose and serum insulin levels. **d** HOMA-IR index. **e** Days 5 and 3: performance of GTTs and ITTs. The two-tailed Student's *t*-test was applied to determine the difference between the group receiving Ad-anti-miR199a-5p and the control group (*P < 0.05, **P < 0.01, ***P < 0.001), *n* = 8 mice per group. **f**–**j** Male C57BL/6J WT mice fed a chow diet were infected with adenovirus-expressing miR199a-5p (Ad-miR199a-5p) or the negative control (Ad-miR NC) via tail vein injection. **f** Day 9: measurement of hepatic miR199a-5p expression. **g, h** Days 6 and 5: examination of fed or fasting blood glucose and serum insulin levels. **i** HOMA-IR index.** j** Days 5 and 3: performance of GTTs and ITTs. The two-tailed Student's *t*-test was applied to determine the difference between the Ad-miR199a-5p group and the control group (*P < 0.05, **P < 0.01, ***P < 0.001), *n* = 6 mice per group

To study the effect of miR199a-5p on insulin sensitivit*y*, we first injected Ad-anti-miR199a-5p adenoviruses or control scrambled adenoviruses (Ad-anti-miR NC) into male HFD-fed C57BL/6J WT mice through the tail vein and then checked the expression of miR199a-5p. Compared with the level in mice that were infected with the control Ad-anti-miR NC viruses, the hepatic miR199a-5p level was significantly inhibited in mice infected with Ad-anti-miR199a-5p viruses, which reduced the miR199a-5p level (Fig. 2a). In comparison with the levels in the control group, the fasting and fed blood glucose levels were significantly lower in Ad-anti-miR199a-5p mice (Fig. 2b). Although the serum insulin level under the fed condition was not affected by Ad-anti-miR199a-5p, a decrease in serum insulin level was observed under the fasting condition in the Ad-anti-miR199a-5p group (Fig. 2c). The decreased fasting blood glucose and insulin levels in the Ad-anti-miR199a-5p group resulted in a lower HOMA-IR index (Fig. 2d), suggesting that knocking down miR199a-5p improved insulin sensitivity, a key factor in regulating glucose homeostasis. We further examined GTTs and ITTs and found that Ad-anti-miR199a-5p mice exhibited reduced glucose tolerance and clearance (Fig. 2e). Because gluconeogenesis is another critical step in regulating hepatic glucose production^28^, we assessed by PTT assay whether miR199a-5p affected gluconeogenesis and checked the expression levels of glucose-6-phosphatase (*G6Pase*) and phosphoenolpyruvate carboxykinase (*PEPCK*)^29^, two key enzymes involved in regulating gluconeogenesis. As shown in Supplementary Fig. 1A-B, miR199a-5p had no effect on gluconeogenesis in the liver, the expression of *G6Pase* or the expression of *PEPCK*, implying that the effect of miR199a-5p mainly occurred through improved insulin sensitivity rather than gluconeogenesis.

### Overexpressed miR199a-5p leads to insulin sensitivity inhibition in vivo

To further explore the role of miR199a-5p in insulin sensitivity, we tried another approach by injecting Ad-miR199a-5p adenoviruses or its control scrambled adenoviruses (Ad-scrambled) into male C57BL/6J WT mice fed normal chow, and detected a significantly higher miR199a-5p level in the mice infected with Ad-miR199a-5p viruses than in mice infected with control viruses (Fig. 2f). Although fed blood glucose levels were not different between the two groups, the fasting blood glucose level was significantly increased in Ad-miR199a-5p mice compared with that in the control mice (Fig. 2g). An increase in the serum insulin level was observed under the fasting condition with miR199a-5p, but not under the fed condition (Fig. 2h). A higher HOMA-IR index in Ad-miR199a-5p mice was also observed due to the elevated fasting blood glucose and insulin levels (Fig. 2i). In addition, Ad-miR199a-5p mice exhibited decreased glucose tolerance and clearance based on GTT and ITT assay data (Fig. 2j). Similarly, the PTT data and the *G6Pase* and *PEPCK* gene expression data indicated that miR199a-5p did not affect gluconeogenesis in the liver (Supplementary Fig. 1C-D).

### miR199a-5p regulates insulin sensitivity by targeting ATG14

To further identify the downstream effector of miR199a-5p, we first predicted potential miR199a-5p mRNA targets by TargetScan, an online bioinformatics tool, and then filtered by conserved binding sites on 8-mer, 7-mer-m8 or 7-mer-A1. A total of 401 transcripts were identified as hits that were conserved in humans and mice. We further filtered these hits based on the following criteria: genes that were reported as target genes of miR199a-5p or genes that were reported as being associated with glucose or lipid metabolism. Using this approach, nine genes were included in our list: *Cav1, Hif1α* and *Ncor1* were reported as target genes of miR199a-5p;^14,30,31^ *Tab3*, *Atg14*, *Sirt1*, *Grb10*, *Map3k5* and *Ucp3* were reported as being associated with glucose or lipid metabolism^9,22,32–35^. To validate the involvement of these genes, their mRNA expression profiles were examined in mice with knocked-down or overexpressed miR199a-5p. Among these nine hits, *Cav1* and *Atg14* were significantly upregulated in mice not expressing miR199a-5p (Fig. 1a), whereas they were downregulated in mice overexpressing miR199a-5p (Fig. 1b). We then focused on *Atg14* because a more robust change in the expression level was detected for *Atg14* than for *Cav1* when the miR199a-5p level was manipulated. Moreover, we are interested in the potential link between autophagy and insulin resistance^17,20,21^, and *Atg14* appears to be the most important regulator involved in autophagic initiation^22^. We then found that the potential miR199a-5p binding site within *Atg14* is highly conserved between humans, mice (Fig. 1c) and other eutherians. To further validate *Atg14* as a target of miR199a-5p, we co-transfected the psiCHECK2-promoter-based *Atg14* 3ʹ-UTR reporter with Ad-miR199a-5p or the negative control into HEK293T cells. We observed that the luciferase activity was significantly suppressed in the group co-transfected with WT *Atg14* and miR199a-5p, but not the group co-transfected with the mutated *Atg14* 3ʹ-UTR reporter and Ad-miR199a-5p (Fig. 1d), suggesting that *Atg14* is a target of miR199a-5p. Thus, we focused on *Atg14* thereafter.

### miR199a-5p regulates autophagy in vivo

**Fig. 3:**
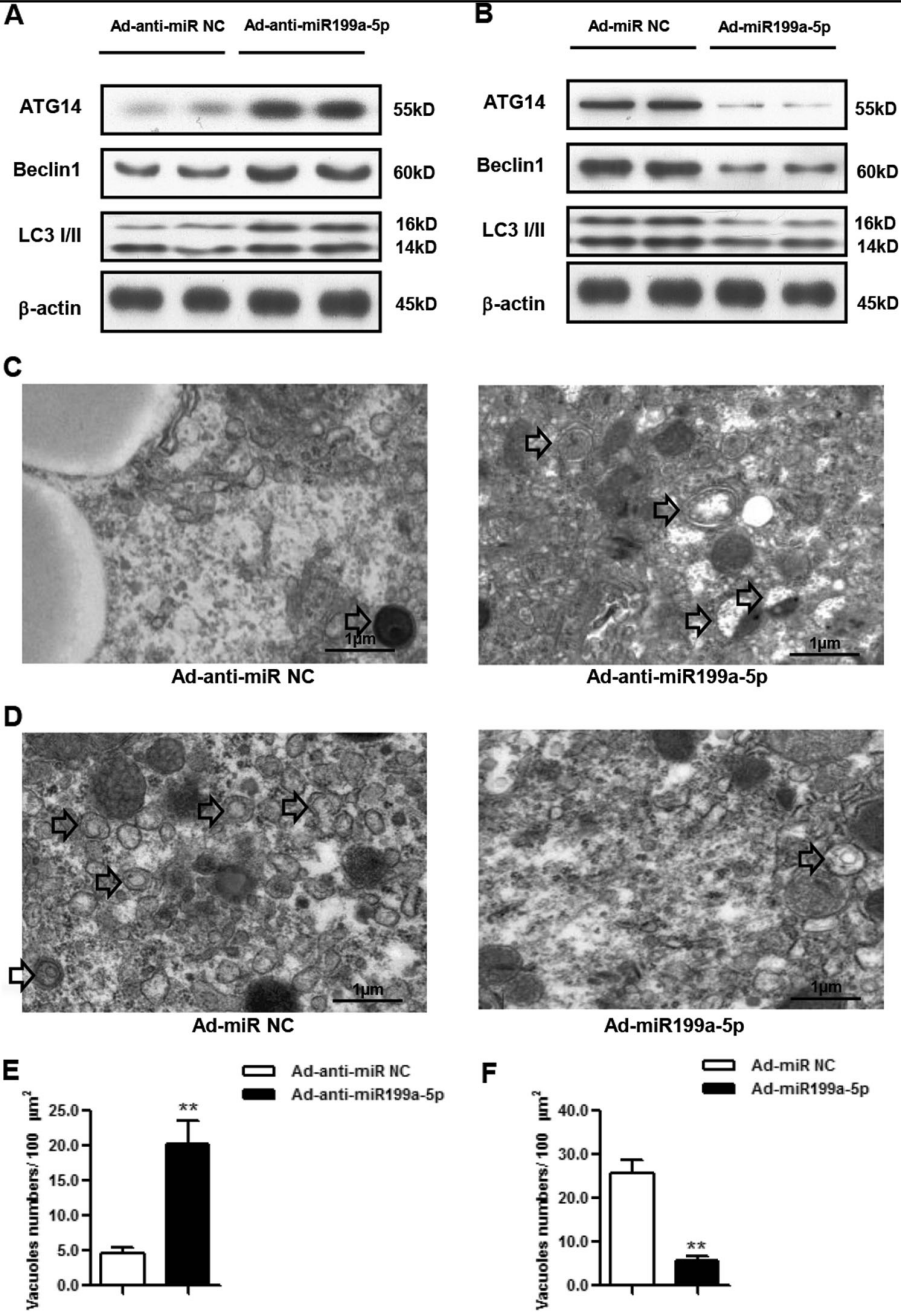
miR199a-5p regulates autophagy in vivo. **a** ATG14, BECLIN1 and LC3 expression levels in male HFD-fed C57BL/6J WT mice expressing miR199a-5p. **b** ATG14, BECLIN1 and LC3 expression levels in male C57BL/6J WT mice overexpressing miR199a-5p. **c** Representative electron microscopic images in male HFD-fed C57BL/6J WT mice expressing miR199a-5p. Arrows indicate autophagosomes/autolysosomes. **d** Representative electron microscopic images in male C57BL/6J WT mice overexpressing miR199a-5p. Arrows indicate autophagosomes/autolysosomes. **e, f** Quantification of autophagosome/autolysosome vacuoles per field in the EM images. The two-tailed Student's *t*-test was applied to determine the difference between the Ad-anti-miR199a-5p group or the Ad-miR199a-5p group and the corresponding control group (**P < 0.01)

To investigate the regulation of autophagy by miR199a-5p in vivo, we measured the protein levels of several autophagy-related proteins, namely ATG14, BECLIN1 and LC3, by taking advantage of the above mice models. We found that the protein levels of ATG14, BECLIN1 and LC3 were increased in HFD mice with knocked-down miR199a-5p (Fig. 3a). Consistently, all three proteins’ levels were decreased in miR199a-5p-overexpressing mice (Fig. 3b). Furthermore, electron microscopic examination of liver samples demonstrated significant increases or decreases in autophagosome/autolysosome formation in mice with inhibited or overexpressed miR199a-5p (Figs. 3c-f), indicating a link between miR199a-5p and autophagy.

### miR199a-5p induces insulin resistance by decreasing ATG14 in vivo

**Fig. 4:**
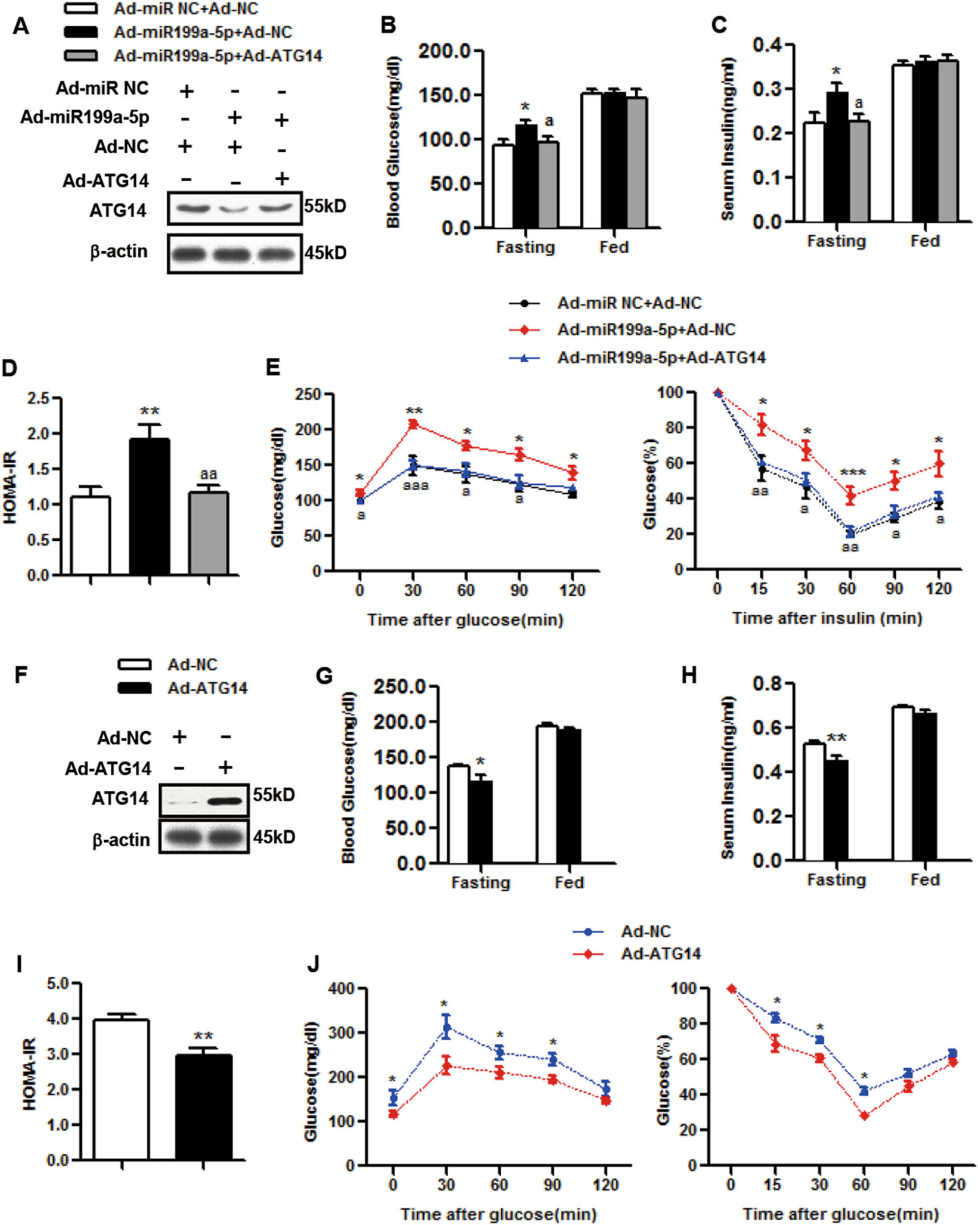
miR199a-5p leads to hepatic insulin resistance via ATG14 in vivo. **a**–**e** Male C57BL/6J WT mice were infected with Ad-miR199a-5p (Ad-miR199a-5p) or the negative control (Ad-miR NC) and with Ad-ATG14 (Ad-ATG14) or the negative control (Ad-NC) via tail vein injection. **a** Day 9: measurement of hepatic ATG14 expression. **b**, **c** Days 6 and 5: examination of fed or fasting blood glucose and serum insulin levels. **d** HOMA-IR index. **e** Days 5 and 3: performance of GTTs and ITTs. The two-tailed Student's *t*-test was applied to determine the difference between the Ad-miR199a-5p group and the corresponding control group (*P < 0.05, **P < 0.01, ***P < 0.001) or between the Ad-miR199a-5p group with Ad-ATG14 and the Ad-miR199a-5p group without Ad-ATG14. (^a^*P* < 0.05, ^aa^*P* < 0.01, ^aaa^*P* < 0.001), *n* = 6 mice per group. **f**–**j**: Male C57BL/6J WT mice were infected with Ad-ATG14 or Ad-NC via tail vein injection. **f** Day 9: measurement of hepatic ATG14 expression. **g**, **h** Days 6 and 5: examination of fed or fasting blood glucose and serum insulin levels. **i** HOMA-IR index. **j** Days 5 and 3: performance of GTTs and ITTs. The two-tailed Student's *t*-test was applied to determine the difference between the Ad-ATG14 group and the corresponding control group (*P < 0.05, **P < 0.01), *n* = 8 mice per group

To gain more insights into the connection between ATG14 and miR199a-5p in the regulation of insulin sensitivity, we injected mice with Ad-miR199a-5p with or without Ad-ATG14. We first examined the effects of Ad-miR199a-5p on ATG14 protein levels in mice, and found that the ATG14 expression levels were decreased by Ad-miR199a-5p (Fig. 4a) but could be partially rescued by the overexpression of Ad-ATG14 (Fig. 4a). Although the overexpression of Ad-ATG14 had no effects on the fed blood glucose or serum insulin levels (Figs. 4b, c), it increased the depressed levels of fasting blood glucose, fasting serum insulin and the HOMA-IR index caused by Ad-miR199a-5p back to normal levels (Figs. 4b–d). In addition, Ad-ATG14 markedly inhibited miR199a-5p-potentiated glucose and insulin tolerance (Fig. 4e). Notably, overexpression of ATG14 alone improved insulin sensitivity in vivo (Figs. 4f–j), suggesting a role of autophagy in insulin resistance.

### miR199a-5p regulates autophagy and autophagic flux in vitro

**Fig. 5:**
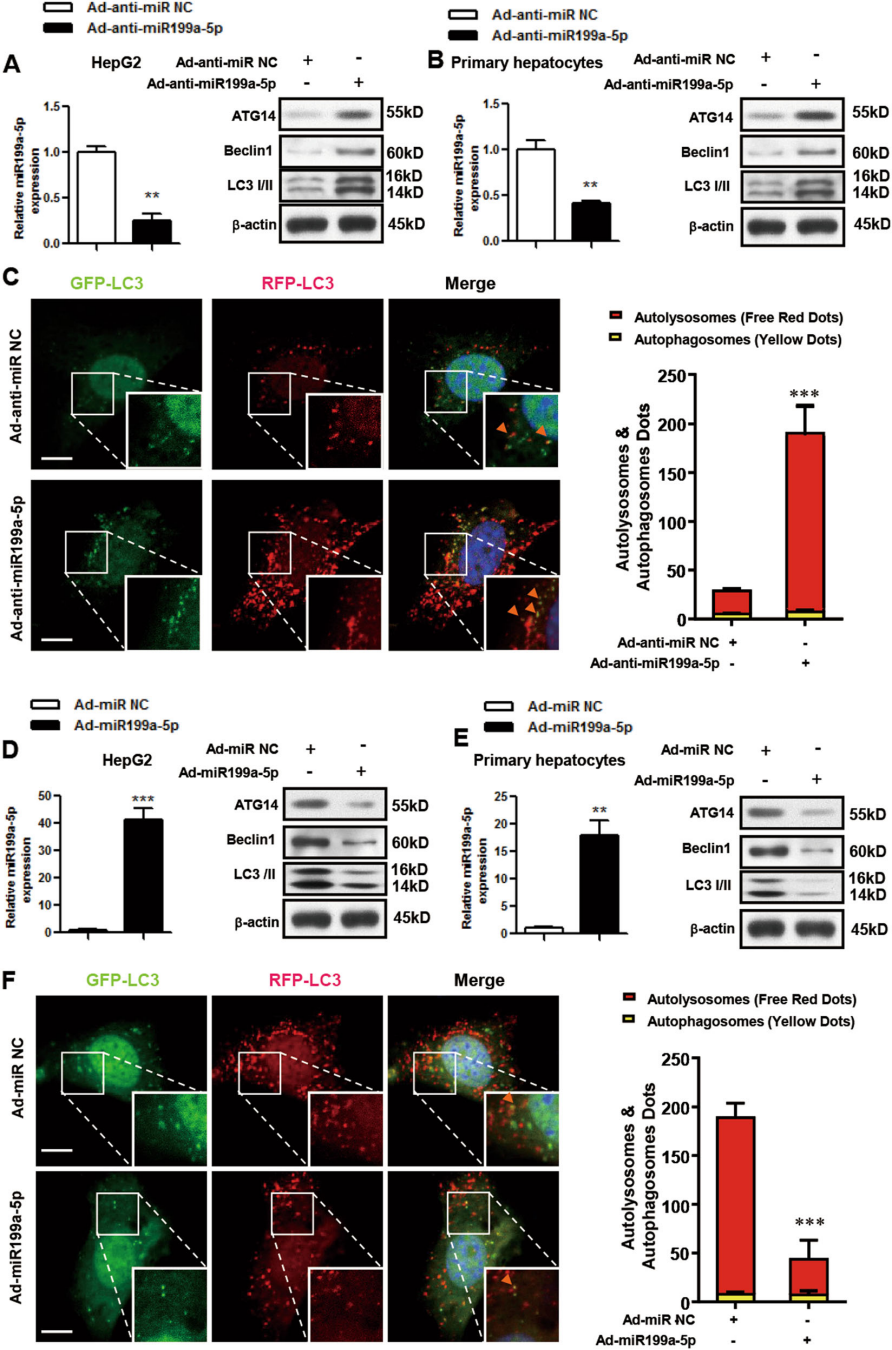
miR199a-5p regulates autophagy in vitro. **a**-**c** HepG2 cells and primary hepatocytes that originated from male HFD-fed C57BL/6J WT mice were infected with adenovirus-inhibiting miR199a-5p (Ad-anti-miR199a-5p) or the negative control (Ad-anti-miR NC) for 48 h. **d-f** HepG2 cells and primary hepatocytes that originated from male C57BL/6J WT mice were infected with adenovirus-expressing miR199a-5p (Ad-miR199a-5p) or the negative control (Ad-miR NC) for 48 h. The expression level of miR199a-5p and the protein levels of ATG14, BECLIN1 and LC3 in HepG2 cells and primary hepatocytes are shown in **a**, **b** and **d,** **e**, respectively. Primary hepatocytes were co-infected with adenoviruses to express mCherry and GFP double-tagged LC3, and the formation of autophagosomes (yellow) and autolysosomes (red) during the autophagy process were analyzed by fluorescence microscopy (**c,** **f**, scale bar: 20 µm, the orange arrows indicate the yellow dots). The two-tailed Student's* t*-test was applied to determine the difference between the Ad-anti-miR199a-5p group or the Ad-miR199a-5p group and the corresponding control group (**P < 0.01, ***P < 0.001)

To explore the regulation of autophagy by miR199a-5p in vitro, we infected HepG2 cells or primary hepatocytes with Ad-anti-miR199a-5p viruses and found that the miR199a-5p level was decreased in these cells. Interestingly, ATG14, BECLIN1 and LC3 protein levels were increased (Figs. 5a, b), highlighting the association between miR199a-5p and the autophagy pathway. To further assess the effect of miR199a-5p on the dynamics of autophagy, we co-infected primary hepatocytes originating from male HFD-fed C57BL/6J WT mice with adenoviruses expressing mCherry and GFP double-tagged LC3 and Ad-anti-miR199a-5p. Because the GFP tag is acid sensitive while the mCherry tag is acid insensitive, both tags emit fluorescent light in autophagosomes, but the green fluorescence from GFP is lost in acidic autolysosomes. This approach allowed us to differentiate autophagosomes (shown as yellow, because red and green colocalize in the cells) versus autolysosomes (shown in red)^27^, and therefore it was employed to monitor autophagic flux in primary hepatocytes. As shown in Fig. 5c, Ad-anti-miR199a-5p promoted a marked increase in autolysosomes, which was consistent with western blot data (Figs. 5a, b).

On the other hand, after overexpressing miR199a-5p in HepG2 cells or primary hepatocytes by Ad-miR199a-5p viruses (Figs. 5d, e), we detected lower protein levels of ATG14, BECLIN1 and LC3 (Figs. 5d, e) and decreased autophagic flux in primary hepatocytes originating from male C57BL/6J WT mice (Fig. 5f), suggesting that miR199a-5p regulates autophagy and autophagic flux via ATG14.

### miR199a-5p inhibits insulin sensitivity by suppressing ATG14 in vitro

**Fig. 6:**
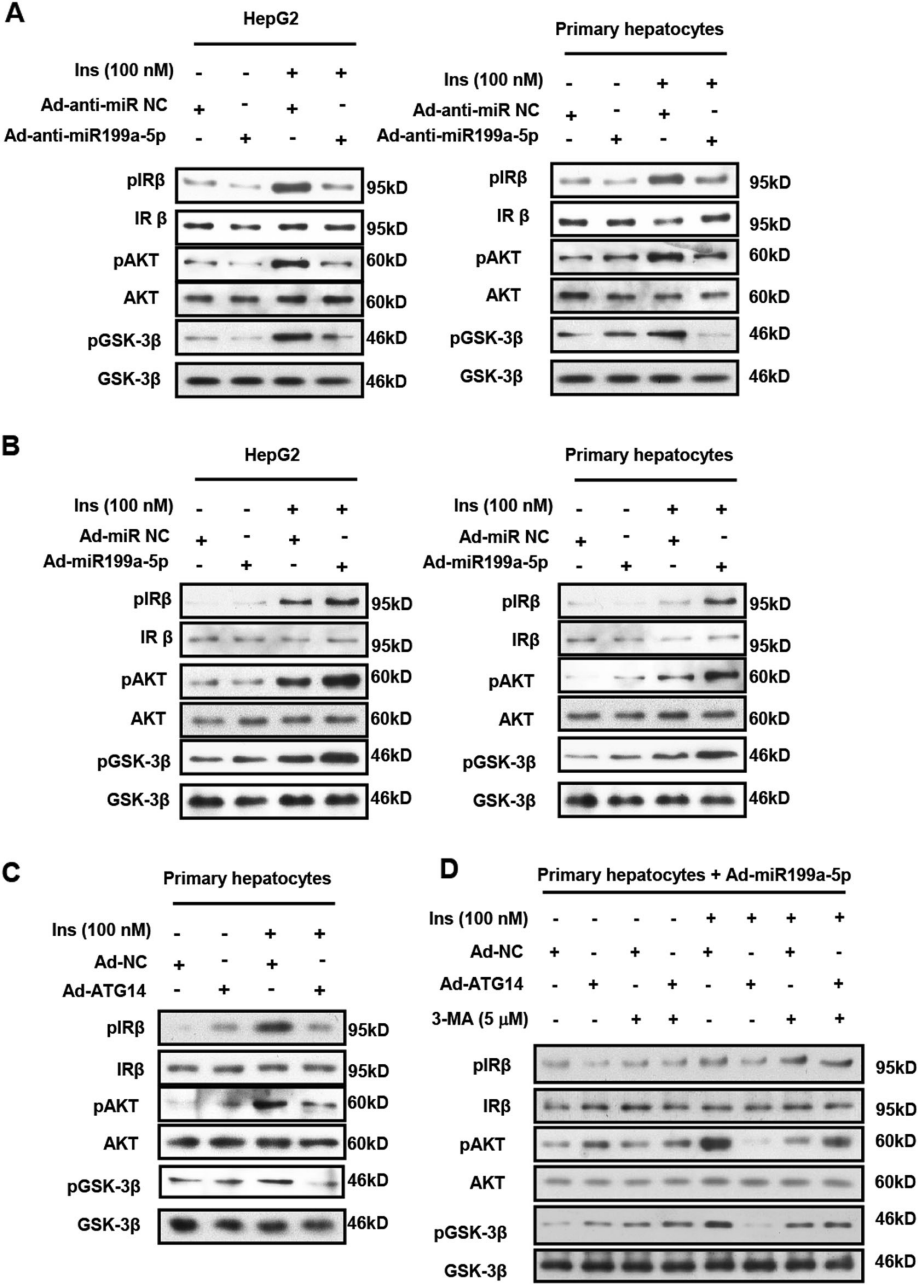
miR199a-5p leads to insulin resistance via ATG14 in vitro. **a** HepG2 cells and primary hepatocytes were infected with adenovirus-expressing miR199a-5p (Ad-anti-miR199a-5p) or the negative control (Ad-anti-miR NC) for 48 h. **b** HepG2 cells and primary hepatocytes were infected with adenovirus-expressing miR199a-5p (Ad-miR199a-5p) or the negative control (Ad-miR NC) for 48 h. **c** Primary hepatocytes were infected with adenovirus-expressing ATG14 (Ad-ATG14) or the negative control (Ad-NC) for 48 h. **d** Primary hepatocytes were infected with adenoviruses expressing miR199a-5p (Ad-miR199a-5p) and ATG14 (Ad-ATG14) or the negative control (Ad-NC) for 48 h, and then treated with 5 μM 3-MA for 2 h. All cases were followed with or without 100 nmol/L insulin (Ins) stimulation for 20 min. p-IRβ (tyr1162/1163), p-AKT (ser473) and p-GSK3β (ser9) protein levels and the total protein level were examined by western blot. Data shown are representative of three independent experiments

To explore the role of miR199a-5p in the regulation of insulin sensitivity in vitro, we assessed insulin signaling by examining insulin-stimulated phosphorylation of IRβ (Tyr1162/1163), AKT (Ser473) and GSK3β (Ser9)^36^. In HepG2 cells and primary hepatocytes with knocked-down or overexpressed miR199a-5p, the phosphorylation of the three key components of insulin signaling was significantly decreased or increased, respectively (Figs. 6a, b), indicating improved and diminished insulin sensitivity, respectively. To gain more insights into the importance of ATG14 in the regulation of insulin sensitivity, we infected primary hepatocytes with Ad-ATG14 viruses to cause the overexpression of ATG14 and found that insulin-induced phosphorylation of IRβ, AKT and GSK3β was significantly decreased in those cells (Fig. 6c), supporting the idea that miR199a-5p regulates insulin sensitivity through ATG14 in vitro. Furthermore, we observed that ATG14 and insulin-induced phosphorylation of IRβ, AKT and GSK3β could be partially reversed by 3-MA, an autophagy inhibitor (Fig. 6d), supporting the relationship between autophagy and hepatic insulin sensitivity.

### Aberrant miR199a-5p/ATG14/hepatic insulin sensitivity axis in diabetes patients

**Fig. 7:**
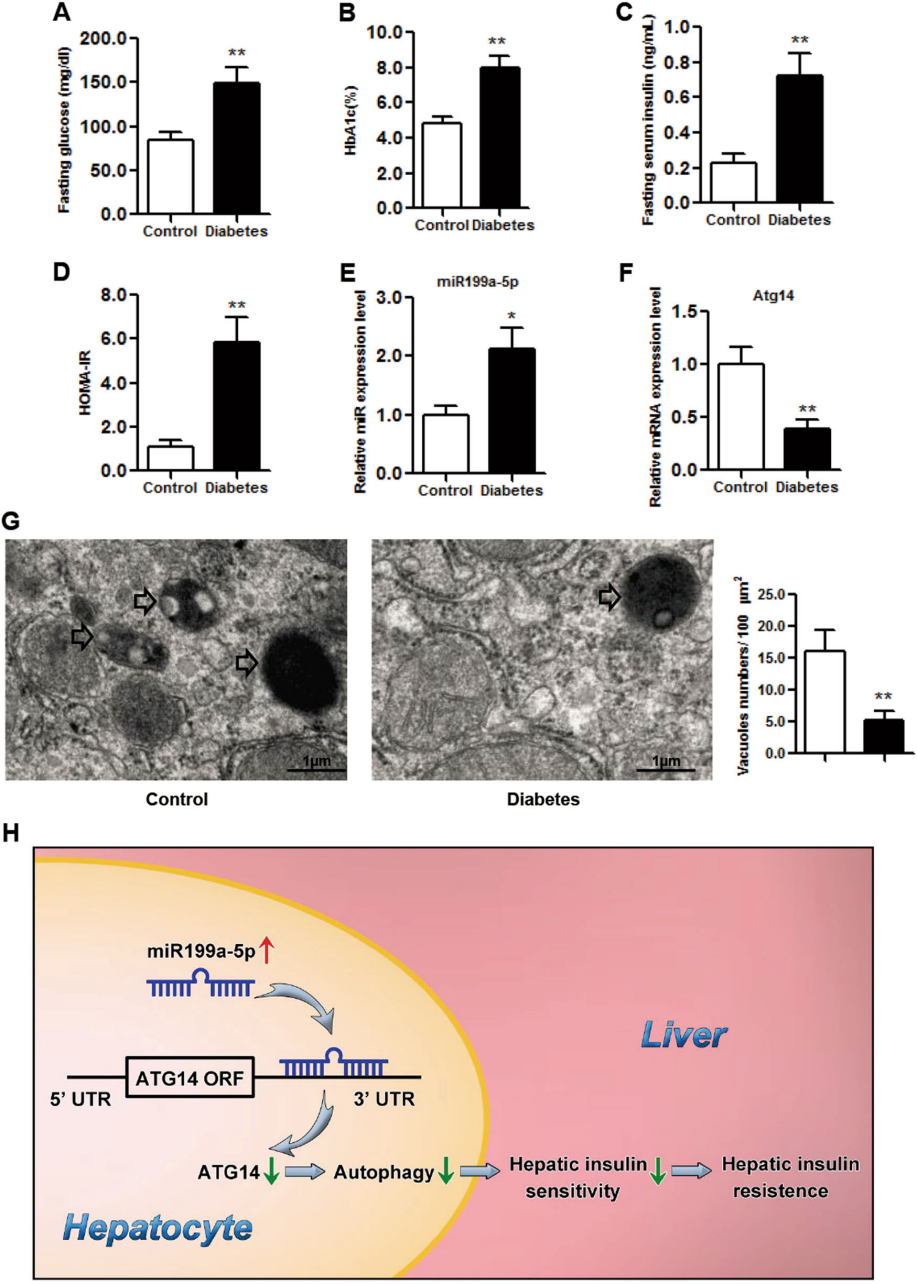
The expression profile of miR199a-5p in the livers of diabetes patients. **a**-**d** The levels of fasting glucose, HbA1c, fasting insulin and HOMA-IR in diabetes patients. **e**, **f** The expression levels of miR199a-5p and *Atg14*. **g** Representative electron microscopic images and quantification of autophagosome/autolysosome vacuoles per field in the EM images in diabetes patients. Arrows indicate autophagosomes/autolysosomes. The two-tailed Student's *t*-test was applied to determine the difference between the diabetes group and the control group, *P < 0.05, **P < 0.01, *t*-test, *n* = 8. **h** Schematic model in which miR199a-5p inhibits hepatic insulin sensitivity via suppressing ATG14-mediated autophagy. We propose that miR199a-5p inhibits ATG14-mediated autophagy, suppresses hepatic insulin sensitivity and eventually leads to insulin resistance, which is a key diabetogenic factor

Finally, we explored the clinical relevance of miR199a-5p by examining miR199a-5p expression levels in liver samples derived from diabetes patients. As expected, the levels of fasting glucose, HbA1c, fasting serum insulin and HOMA-IR were markedly elevated in these diabetes patients (Figs. 7a-d). We also detected upregulated miR199a-5p (Fig. 7e) and suppressed *Atg14* mRNA levels (Fig. 7f) in these patients, which was consistent with our hypothesis. Furthermore, TEM data demonstrated a significant reduction in autophagosome/autolysosome formation in diabetes patients (Fig. 7g), strongly supporting a correlation between miR199a-5p/ATG14-mediated autophagy and hepatic insulin sensitivity in diabetes patients.

## Discussion

It has been reported that miR199a-5p contributes to lung fibrotic processes, embryonic stem cell-derived hepatic cell repopulation, liver cancer glucose metabolism^10–13^ and hepatic steatosis^14^. Because hepatic insulin resistance is closely related to fatty liver, we tested the hypothesis that miR199a-5p regulates hepatic insulin sensitivity^15,16^. We first showed that knocking down miR199a-5p resulted in decreased glucose tolerance and clearance in vivo and that overexpression of miR199a-5p had the opposite effect. These effects could be partially rescued by overexpressing ATG14. Second, knocking down miR199a-5p led to decreased insulin-induced phosphorylation of IRβ, AKT and GSK3β in vitro, and overexpression of miR199a-5p potentiated their phosphorylation. Importantly, we provided evidence of increased hepatic miR199a-5p levels in diabetes patients, which correlated with decreased ATG14 levels and autophagy. To our knowledge, this study is the first report to illustrate a novel biologic function of miR199a-5p in the regulation of hepatic insulin sensitivity.

Mature miR199a-5p is a molecule 21 nucleotides in length with several proteins as downstream targets^14,30,31^. To fully uncover the molecular mechanism by which miR199a-5p regulates insulin sensitivity, we profiled the expression levels of potential target genes of miR199a-5p, which had been reported as target genes of miR199a-5p or as being associated with glucose or lipid metabolism^9,22,32–35^, and identified ATG14 as a target of miR199a-5p. ATG14 is the most important regulator involved in autophagic initiation^22^. A previous study reported that ectopic overexpression of ATG14 resulted in lipophagy-mediated stress^37^, and another study showed that the gain and loss of function of ATG14 resulted in the fall and rise in triglyceride levels in mice liver, respectively, emphasizing its critical role in hepatic lipophagy^22^. Our data supported that ATG14 is a direct target of miR199a-5p and that its role in the miR199a-5p cascade caused insulin resistance.

Several reports have demonstrated the relationship between autophagy and insulin resistance^17–21^. For example, in the livers of mice with hyperinsulinemia and insulin resistance, autophagy was inhibited, and the expression levels of several key autophagy genes were also suppressed^17^. The reduced expression of Atg7 in the liver induced by adenovirus-mediated delivery of shAtg7 leads to increased endoplasmic reticulum stress and worsened insulin resistance in lean mice, whereas the adenovirus-mediated overexpression of WT Atg7 relieves these features in obese mice^18^. Similarly, the adenovirus-mediated overexpression of transcription factor EB, a master regulator of lysosomal biogenesis and autophagy, ameliorates insulin resistance in mice fed a HFD or in ob/ob mice^19^. Some drugs that are known to activate autophagy (such as carbamazepine and rapamycin) can improve insulin homeostasis^20^. In addition, the protective effect of some non-autophagy-specific drugs against hepatosteatosis and insulin resistance in obese mice has been linked to the activation of autophagy by these drugs^21^. Our study also found a critical relationship between hepatic autophagy and insulin resistance. More importantly, we demonstrated that in type 2 diabetes patients and animal models the hepatic miR199a-5p level is upregulated, whereas the ATG14 level and autophagy levels are suppressed, which implies the importance of the miR199a-5p/ATG14 axis in translational diabetes research.

The miR199a-5p sequence is highly conserved in humans and mice. miR199a-1 and miR199a-2 are precursors for miR199a-5p, and evolutionary studies have found that the predominant expression patterns of miR199a-1 and miR199a-2 in tissues surrounding developing craniofacial skeletal elements are consistent between humans and mice, indicating a conserved role of miR199a-1 and miR199a-2 in vertebrate skeletogenesis^38^ and implying the potential translational application of miR199a-5p from mice to humans in the future.

Our previous study unveiled the critical role of CAV1 in hepatic steatosis^14^, and another study reported that CAV1-deficient mice showed insulin resistance in adipose tissue^39^. In the current study, we also found that hepatic CAV1 was upregulated or downregulated in miR199a-5p knocked-down or overexpressing mice, respectively. Because the change in CAV1 levels was not as robust as in ATG14 levels, we focused on ATG14 in the current study. However, we cannot rule out the possibility that the effect of miR199a-5p on the regulation of hepatic insulin sensitivity may be partially mediated by CAV1 in mice. Our previous work found that miR199a-5p/hepatic steatosis could be regulated by free fatty acids (FFAs)^14^, and other reports have also suggested a potential link between hepatic insulin resistance and hepatic steatosis^15,16^. Thus, miR199a-5p may also be regulated by FFAs, a potential link that needs to be investigated in the future. Although the approach of adenovirus infection is not as clean as that of hepatic conditional knockout mice, it is believed that the adenovirus-mediated infection mainly occurs through the liver, and it has been widely utilized in previous hepatic research^9,24^.

In conclusion, our data provide strong evidence to support the role of miR199a-5p in the regulation of hepatic insulin sensitivity. Furthermore, we uncovered a novel mechanism whereby dysregulated miR199a-5p suppresses the expression of ATG14 and inhibits autophagy, which in turn leads to the downregulation of hepatic insulin sensitivity and eventually causes insulin resistance (Fig. 7h). Our study also demonstrated the significance of the miR199a-5p/ATG14 axis in translational diabetes research, which may serve as a potential therapeutic target for hepatic insulin sensitivity and other associated metabolic diseases.

## Electronic supplementary material

supplementary material(PDF 368 kb)
