# Supplementary material for: miR199a-5p inhibits hepatic insulin sensitivity via suppression of ATG14-mediated autophagy
Source: Cell Death Dis. 2018 Mar 14;9(3):405. doi: 10.1038/s41419-018-0439-7 (PMC5851987; doi:10.1038/s41419-018-0439-7)
Supplement: Supplementary file 1 — supplementary material(PDF 368 kb) [file 41419_2018_439_MOESM1_ESM.pdf]

Figure S1.

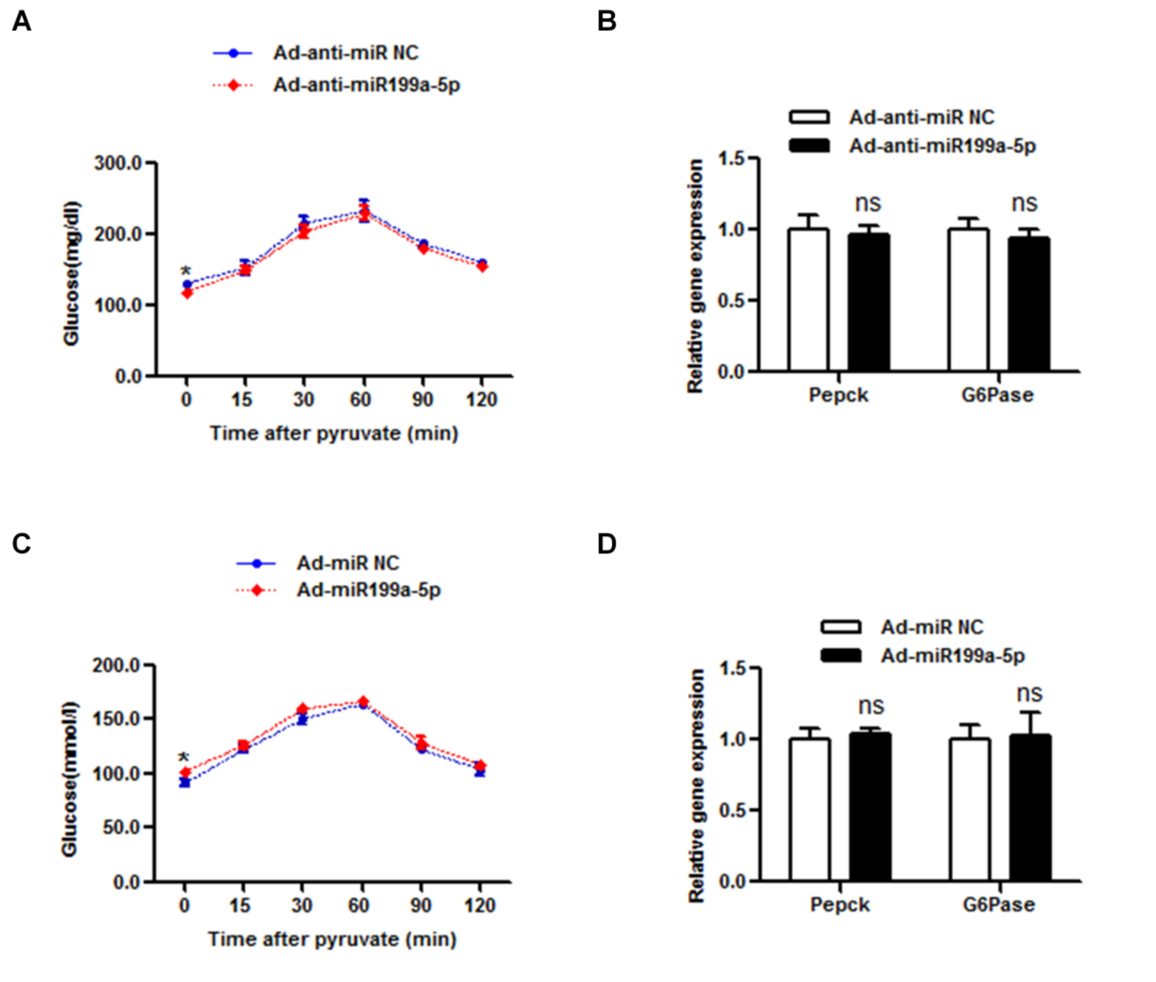

Figure legends.

Figure S1. Inhibition or overexpression of miR199a-5p has no effect on gluconeogenesis *in vivo*.

A-B: Male C57BL/6J WT mice were fed with high fat diet for 3 months, then infected with adenovirus-inhibiting miR199a-5p (Ad-anti-miR199a-5p) or negative control (Ad-anti-miR NC) via tail-vein injection. A. Performance of PTTs at day 7. B. mRNA expression levels of *PEPCK* and *G6Pase*. The two-tailed Student t test was applied to determine the difference between the Ad-anti-miR199a-5p group and the control group (*P < 0.05, ns, not statistically significant),

n=8 per group. C-D: Male C57BL/6J WT mice were infected with adenovirus-expressing miR199a-5p (Ad-miR199a-5p) or negative control (Ad-miR NC) via tail-vein injection. C. Performance of PTTs at day 7. D. mRNA expression levels of *Pepck* and *G6Pase*. The two-tailed Student t test was applied to determine the difference between the Ad-anti-miR199a-5p group and the control group (*P < 0.05, ns, not statistically significant), n=6 per group.

Supplementary Table 1.

Primers used for real-time quantitative PCR.

Genes	Forword primer(5' -> 3')	Reverse primer(5' -> 3')
Atg14	CACTGCACACACTCAGGAAC	ATCTCCACTCGCGTCTGATT
Cav1	GCTCTGCCCGGGTGTGGAAA	AACGGTGTAGAGATGTCCCTGCG
Hif1α	GATGACGGCGACATGGTTTAC	CTCACTGGGCCATTTCTGTGT
Ncor1	TATGGACCGGGTAGACCGAG	CGGTGTTTTTGTTCCACAGGAG
Tab3	ATGGCGCAAAACAGTCCACA	TGTAACATGCACTGGGATACCA
Sirt1	TGATTGGCACCGATCCTCG	CCACAGCGTCATATCATCCAG
Grb10	GTGGTGGAGATTCTAACCGACA	ACCTCTCTAATCCCAGTTGTGG
Map3k5	CGTGCTGGACCGTTTTTACAA	TGTCACCATGTAGGGGATGAAG
Ucp3	CTGCACCGCCAGATGAGTTT	ATCATGGCTTGAAATCGGACC
Pepck	CTGCATAACGGTCTGGACTTC	GCCTTCCACGAACTTCCTCAC
G6Pase	CTGTTTGGACAACGCCCGTAT	AGGTGACAGGGAACTGCTTTA
β-Actin	GGCTGTATTCCCCTCCATCG	CCAGTTGGTAACAATGCCATGT
Atg14*	CGATCACAACGGAGACACCA	CAGCACTGATGGTGTAGGCA
β-Actin*	CATGTACGTTGCTATCCAGGC	CTCCTTAATGTCACGCACGAT
miR199a-5p	GGGCCCAGTGTTCAGACTA	TGCGTGTCGTGGAGTC
U6	CTCGCTTCGGCAGCACA	ACGCTTCACGAATTTGCGT

*human targets.
